# Real-World Indirect Treatment Comparison of Terlipressin vs Midodrine Plus Octreotide in Hepatorenal Syndrome-Acute Kidney Injury

**DOI:** 10.14309/ctg.0000000000000951

**Published:** 2025-11-20

**Authors:** Stevan A. Gonzalez, Andrew S. Allegretti, Viktor V. Chirikov, Wei-Jhih Wang, Xingyue Huang, Douglas A. Simonetto, Kevin Moore

**Affiliations:** 1 Division of Hepatology, Annette C. and Harold C. Simmons Transplant Institute, Baylor Scott & White All Saints Medical Center, Fort Worth, Texas, USA;; 2 Department of Medicine, Burnett School of Medicine at TCU, Fort Worth, Texas, USA;; 3 Department of Medicine, Division of Nephrology, Massachusetts General Hospital, Boston, Massachusetts, USA;; 4 OPEN Health, New York, New York, USA;; 5 Mallinckrodt Pharmaceuticals, Hampton, New Jersey, USA;; 6 Division of Gastroenterology and Hepatology, Mayo Clinic College of Medicine and Science, Rochester, Minnesota, USA;; 7 UCL Institute for Liver and Digestive Health, Royal Free Hospital, University College London, London, UK.

**Keywords:** real-world data, retrospective chart review, vasopressin analog

## Abstract

**INTRODUCTION:**

Evidence on the comparative real-world effectiveness of terlipressin vs midodrine plus octreotide (MO) for hepatorenal syndrome-acute kidney injury (HRS-AKI) in the United Kingdom and the United States is limited.

**METHODS:**

Using individual-level chart review data for patients across the United Kingdom (2013–2017) and the United States (2016–2019), an indirect treatment comparison was conducted comparing the efficacy of terlipressin (UK cohort) with MO (US cohort). Covariate balancing propensity scoring matched the cohorts on baseline serum creatinine (SCr), presence of encephalopathy and/or ascites, albumin use and duration, age, and sex. The primary endpoint was HRS reversal, defined as achieving SCr ≤1.5 mg/dL by the last day of treatment.

**RESULTS:**

At treatment initiation, 90.2% of UK patients received terlipressin (194/215), while 89.2% of US patients received MO (140/157). Concomitant albumin was administered in 67.9% of UK and 98.7% of US patients. In a covariate balancing propensity score-adjusted cohort, HRS reversal was achieved in 53.2% of terlipressin-treated patients (the United Kingdom, weighted effective sample size of 75) compared with 16.9% of MO-treated patients (the United States, n = 89) (adjusted mean difference (95% CI) 36.3% (22.4, 50.2), *P* < 0.0001). In adjusted analysis, individuals treated with terlipressin experienced an overall reduction in SCr at completion of treatment (SCr decrease 1.00 mg/dL vs increase of 0.08 mg/dL for MO-treated patients, *P* < 0.0001).

**DISCUSSION:**

HRS-AKI treatment and outcomes differ between the United Kingdom and the United States, attributed to the historical standard of care MO in the United States. In adjusted analyses, real-world use of terlipressin was more effective than MO at improving kidney function and achieving HRS-AKI reversal.

## Graphical abstract

**Figure:**
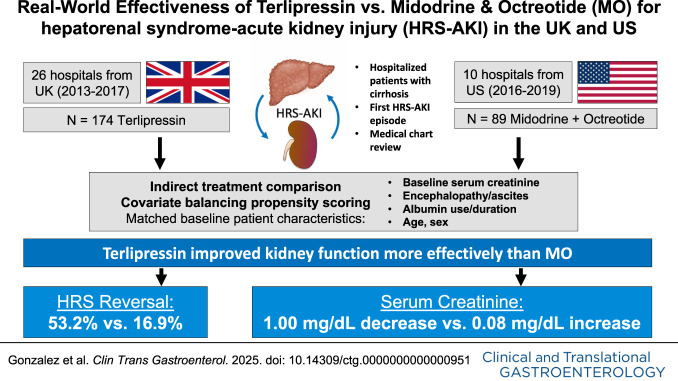

## INTRODUCTION

Hepatorenal syndrome-acute kidney injury (HRS-AKI) is a severe form of AKI that develops in patients with advanced cirrhosis in the absence of another identifiable cause (1) and is characterized by renal vasoconstriction resulting in decreased kidney perfusion and effective arterial volume (2,3). AKI diagnosis and staging are defined by an acute rise in serum creatinine (SCr) and can be classified among individuals with cirrhosis based on etiology, including prerenal, postrenal, acute tubular necrosis, and HRS-AKI (4,5). Although up to 50% of patients with cirrhosis may develop AKI (6), HRS-AKI is relatively rare, accounting for 12%–20% of hospitalized patients with AKI and cirrhosis, as reported in a recently published study with data from 11 US hospital networks (4). In contrast with the majority of individuals with cirrhosis who present with AKI, individuals with HRS-AKI fail to respond to volume repletion in the absence of other causes of functional or structural kidney disease. HRS-AKI is reversible with treatment but if untreated, the consequences of HRS-AKI include irreversible kidney failure, with >80% mortality at 3 months and a median survival of less than 4 weeks (7–9).

Both the 2018 European Association for the Study of the Liver and the 2021 American Association for the Study of Liver Disease guidelines recommend terlipressin, a synthetic vasopressin receptor agonist, in combination with albumin as first-line treatment to improve kidney function in adults with HRS-AKI (2,10). However, although it has been recommended and approved in Europe over 2 decades, terlipressin was only recently approved by the US Food and Drug Administration in 2022 based on the results from the phase 3 randomized, double-blind, placebo-controlled CONFIRM trial (11). In addition, using terlipressin data from the CONFIRM/REVERSE clinical trials, this study team demonstrated in US settings that terlipressin plus albumin improves kidney function in HRS-AKI compared with historic standard of care treatment with midodrine and octreotide (MO) plus albumin (12).

Real-world HRS-AKI patient populations have been previously described in Europe, based on observational data from 26 hospitals in the United Kingdom (13,14), as well as in the United States, based on retrospective chart review data from 10 tertiary care centers (14,15). However, no evidence is available to describe any differences or similarities in HRS-AKI patient characteristics, diagnosis, treatment, and outcomes or real-world comparative data between the 2 countries. In addition, most hospitalized patients in the United States still receive MO (16). Therefore, this study had 2 aims. The first was to describe patient characteristics, initial treatment, and health outcomes using individual-level chart review data among patients with HRS-AKI across the United Kingdom and the United States. The second was to conduct an indirect comparison of the real-world effectiveness of terlipressin vs MO, which were the 2 treatments with the highest use for HRS-AKI (90.2% for the United Kingdom and 89.2% for the United States) in the observational data from the United Kingdom and the United States, respectively.

## METHODS

### Study design and data source

The study was a post hoc analysis of retrospective data abstracted from medical charts of hospitalized patients for first HRS-AKI episode from 26 UK medical centers with a hepatology unit (from January 2013 to December 2017) and 10 US tertiary medical centers with a hepatology and liver transplant (LT) unit (January 2016–December 2019) (13–15). Participating investigators identified hospitalized patients with a clinical diagnosis of HRS-AKI based on expert review and collected data (ie, demographics, clinical characteristics, treatment history, healthcare resource use, and clinical outcomes) from hospital admission to 90-day postdischarge or until death. The 2 retrospective chart reviews used an identical study protocol, except for the collection of additional variables (eg, bilirubin, international normalized ratio, hepatic encephalopathy grade, and respiratory failure) in the US study used for the calculation of Model for End-stage Liver Disease (MELD), Acute on Chronic Liver Failure (ACLF) grade, and Child-Pugh scores. The US study was approved by central and local Institutional Review Boards (IRB), including the Western IRB/Copernicus Group IRB. The UK study used a decision tool created by the UK Health Research Authority, which granted a waiver for obtaining informed consent.

### Study population

Eligible adult patients were identified using *International Classification of Diseases, 9th Revision* (*ICD-9*) or *10th Revision* (*ICD-10*), codes and/or based on the documentation of HRS-AKI diagnosis (subject to individual investigator's clinical judgment, including capture of primary reason for hospital admission, International Club of Ascites HRS criteria met, setting of diagnosis, and precipitating events) within the medical chart. Diagnosis of HRS-AKI was confirmed by expert review or principal investigator at each site. Patients were excluded if they were enrolled in any clinical trial during hospitalization, had incomplete laboratory data for assessment of treatment response, or had a hospital stay ≤2 days. Further details on the UK and US chart review studies have been previously published and presented (12–14).

For the first study objective, the original sample of N = 250 for UK and N = 200 for US chart review data was restricted to N = 215 and N = 157, respectively, to achieve consistency by keeping only those patients who had measured SCr at baseline and follow-up, had SCr value ≥ 1.5 mg/dL at the time of hospitalization for HRS-AKI, initiated either terlipressin or standard of care (including MO, norepinephrine, and vasopressin). Specifically for the US cohort, patients did not have receipt of renal replacement therapy (RRT), LT, or transjugular intrahepatic portosystemic shunt (TIPS) within 2 days of treatment initiation. The last condition regarding the relative timing of RRT/LT/TIPS receipt could not be implemented for the UK cohort as the dates for the administration of those procedures were not captured in the UK chart review data.

For the second objective, a subcohort of patients, derived from the patient sample in the previous objective, that was treated for at least 2 days with terlipressin (N = 174 from the United Kingdom) or MO (N = 89 from the United States) was included to assess the real-world HRS-AKI outcomes between the terlipressin and the MO (Figure 1). In addition, midodrine and octreotide had to be used within 2 days of each other to be considered an MO combination, while those who switched to norepinephrine as subsequent treatment and used it for more than 1 day were excluded.

Figure 1.Patient attrition flowchart. (**a**) Overall cohort: UK and US chart review. (**b**) Development of covariate balancing propensity score cohort: Terlipressin (the United Kingdom) vs midodrine plus octreotide (the United States). LT, liver transplantation; RRT, renal replacement therapy; SCr, serum creatinine; TIPS, transjugular intrahepatic portosystemic stent shunt; TX, treatment.

**Figure:**
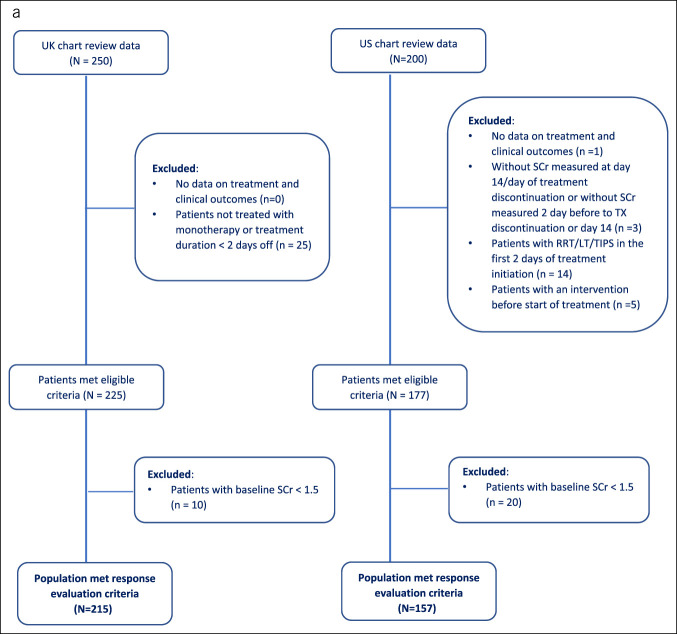

**Figure:**
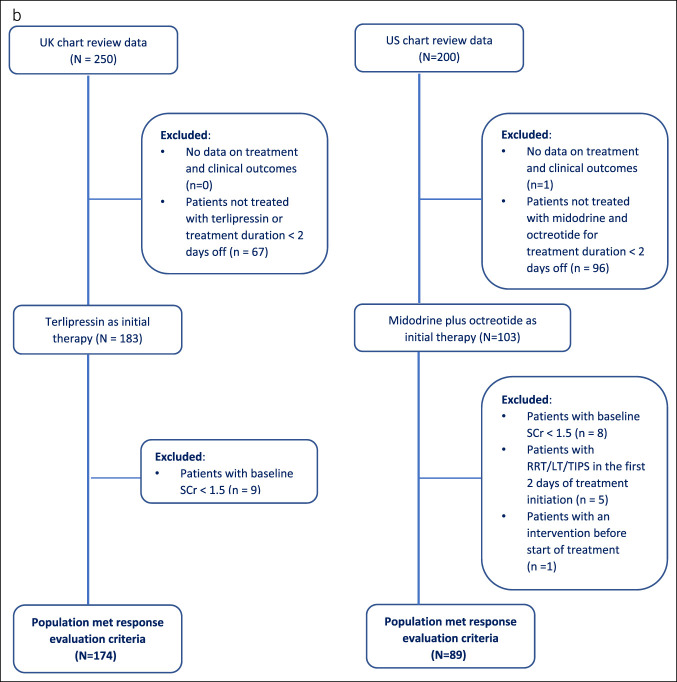

### Statistical analysis and outcomes

#### Unadjusted descriptive analysis of the UK and US cohorts

Patient characteristics and clinical outcomes were summarized descriptively for the UK and US cohorts. HRS-AKI treatment response was measured up to 14 days (or day of treatment discontinuation) and defined as follows: (i) HRS reversal: decrease in SCr from baseline to a final level of ≤1.5 mg/dL; (ii) partial response: ≥30% decrease in SCr from baseline to a final level >1.5 mg/dL; and (iii) no response: <30% decrease in SCr from baseline. If another intervention such as RRT or LT were initiated during the vasoconstrictor drug therapy time window of up to 14 days, then only SCr values up until the date before RRT/LT treatment were considered as part of calculating HRS-AKI response (these data were only available for US cohort). Further investigation was conducted on the effect of RRT vs non-RRT patients on SCr values, with the conclusion that this limitation would not materially bias the results for the UK cohort as described in the RRT section of the Results, as well as in the Discussion. Additional outcomes analysis included on-treatment HRS reversal status, defined as achieving SCr ≤1.5 mg/dL at any time during the treatment window, as well as absolute and relative changes in SCr since baseline. Overall survival (OS) was defined as survival time from treatment start date to death and reported over follow-up of 90 days.

#### Adjusted indirect real-world treatment comparison between terlipressin and MO

The primary outcome for the second study objective was the difference in HRS-AKI treatment response between terlipressin and MO (ie, HRS reversal). Secondary outcomes included on-treatment HRS reversal (defined as achieving SCr ≤1.5 mg/dL at any time during the treatment window), partial response at the last day of treatment, mean SCr changes from baseline, and OS.

The following clinically relevant characteristics were selected for adjustment: age, sex, baseline SCr severity (mild/moderate/severe), presence of encephalopathy or ascites at the time of hospitalization, and albumin use and duration during the HRS-AKI hospitalization. Other characteristics such as sodium, bilirubin, MELD, Child-Pugh, and ACLF grade could not be adjusted for because they were available only in the US chart data. As formal MELD, Child-Pugh, and ACLF grade could not be used for the adjusted indirect comparison between the UK and US cohorts, the presence of encephalopathy and presence of ascites at the time of hospitalization were considered surrogates for the severity of the clinical presentation among study cohorts; therefore, those 2 patient characteristics were selected for adjustment.

The adjusted indirect comparison was performed using covariate balancing propensity score (CBPS)-weighted methodology (17). Using the weights from CBPS, terlipressin-treated patients were statistically reweighted to those from the MO cohort as a reference. Weighted means and t-tests were used to compare response-related and SCr-related outcomes between the terlipressin and MO patient cohort, while weighted Cox proportional hazards model and Kaplan-Meier curves were used to compare OS.

## RESULTS

### Comparison of UK and US cohorts

A total of 215 and 157 patients were identified from the UK and US cohorts, respectively (Table 1). Patients in the UK cohort were younger than in the US cohort (median age 54 vs 59 years) and had numerically higher average SCr values at baseline (3.34 mg/dL vs 3.00 mg/dL, *P* = 0.10). Among individuals with SCr ≥5 mg/dL, UK patients (N = 22) had higher average baseline SCr levels than US patients (N = 13) (7.07 mg/dL vs 5.78 mg/dL, *P* < 0.01). Overall, patients in the United Kingdom also had a lower proportion of encephalopathy (33% vs 60.5%) and ascites (74.4% vs 92.4%) at hospital admission than in the United States. Alcohol-related cirrhosis was the leading etiology in both the United Kingdom (68.4%) and the United States (52.9%). The most reported precipitating events in the United Kingdom and the United States were treatment with diuretics (36.7% vs 42.7%), followed by gastrointestinal bleeding in the United Kingdom (27.9%) and large-volume paracentesis (42.0%) in the United States.

**Table 1.** Baseline and clinical characteristics among patients with HRS-AKI in the United Kingdom and the United States

Variable	Statistic or category	All patients			Mild (≤3 mg/dL)			Moderate (>3 & <5 mg/dL)			Severe (≥5 mg/dL)		
		The United Kingdom	The United States	*P* value	The United Kingdom	The United States	*P* value	The United Kingdom	The United States	*P* value	The United Kingdom	The United States	*P* value
		(N = 215)	(N = 157)		(N = 105)	(N = 94)		(N = 88)	(N = 50)		(N = 22)	(N = 13)	
Initiation treatment, N (%)	Dobutamine	0 (0.0%)	1 (0.6%)	**<0.001** ^**C**^	0 (0.0%)	1 (1.1%)	**<0.001** ^**E**^	0 (0.0%)	0 (0.0%)	**<0.001** ^**E**^	0 (0.0%)	0 (0.0%)	**<0.001** ^**E**^
	Midodrine/octreotide	4 (1.9%)	140 (89.2%)		1 (1.0%)	83 (88.3%)		3 (3.4%)	46 (92.0%)		0 (0.0%)	11 (84.6%)	
	Norepinephrine	4 (1.9%)	6 (3.8%)		3 (2.9%)	3 (3.2%)		1 (1.1%)	3 (6.0%)		0 (0.0%)	0 (0.0%)	
	Other/combination therapy	0 (0.0%)	10 (6.4%)		0 (0.0%)	7 (7.4%)		0 (0.0%)	1 (2.0%)		0 (0.0%)	2 (15.4%)	
	Terlipressin	194 (90.2%)	0 (0.0%)		101 (96.2%)	0 (0.0%)		71 (80.7%)	0 (0.0%)		22 (100.0%)	0 (0.0%)	
	Vasopressin	13 (6.0%)	0 (0.0%)		0 (0.0%)	0 (0.0%)		13 (14.8%)	0 (0.0%)		0 (0.0%)	0 (0.0%)	
Time to initial treatment	Median (Q1–Q3)	4.0 (2.0–8.0)	2.0 (1.0–4.0)	**<0.001**	4.0 (2.0–8.0)	2.0 (1.0–5.0)	**0.004**	4.0 (3.0–7.5)	2.0 (1.0–4.0)	**<0.001**	4.0 (2.0–9.0)	2.0 (1.0–3.0)	**0.037**
Age at admission (yr)	Median (Q1–Q3)	54.0 (46.0–62.0)	59.0 (50.0–66.0)	**0.001** ^**W**^	55.0 (45.0–63.0)	57.0 (49.0–66.0)	0.150^W^	54.0 (46.5–62.0)	61.5 (51.0–67.0)	**0.002** ^**TE**^	53.0 (46.0–61.0)	60.0 (49.0–64.0)	0.231^W^
Sex, N (%)	Male	146 (67.9%)	87 (55.4%)	**0.014** ^**C**^	65 (61.9%)	52 (55.3%)	0.346^C^	64 (72.7%)	25 (50.0%)	**0.007** ^**C**^	17 (77.3%)	10 (76.9%)	>0.999^E^
	Female	69 (32.1%)	70 (44.6%)		40 (38.1%)	42 (44.7%)		24 (27.3%)	25 (50.0%)		5 (22.7%)	3 (23.1%)	
SCr level in mg/dL at baseline	Mean (SD)	3.34 (1.62)	3.00 (1.19)	0.098^W^	2.18 (0.37)	2.22 (0.40)	0.504 ^TE^	3.79 (0.53)	3.76 (0.54)	0.807^W^	7.07 (1.62)	5.78 (0.65)	**0.002** ^**TU**^
	Median (Q1–Q3)	3.1 (2.1–4.0)	2.7 (2.1–3.6)		2.1 (1.9–2.4)	2.2 (1.9–2.6)		3.7 (3.4–4.2)	3.6 (3.3–4.3)		6.6 (5.8–8.8)	5.5 (5.3–6.1)	
	Range	1.5 to 10.6	1.5 to 7.3		1.5 to 3.0	1.5 to 3.0		3.1 to 5.0	3.1 to 4.9		5.0 to 10.6	5.0 to 7.3	
Severity (in mg/dL), N (%)	Mild (**≤**3)	105 (48.8%)	94 (59.9%)	0.108^C^	105 (100.0%)	94 (100.0%)	NA	0 (0.0%)	0 (0.0%)	NA	0 (0.0%)	0 (0.0%)	NA
	Moderate (>3 & <5)	88 (40.9%)	50 (31.8%)		0 (0.0%)	0 (0.0%)		88 (100.0%)	50 (100.0%)		0 (0.0%)	0 (0.0%)	
	Severe (≥5)	22 (10.2%)	13 (8.3%)		0 (0.0%)	0 (0.0%)		0 (0.0%)	0 (0.0%)		22 (100.0%)	13 (100.0%)	
Presence of encephalopathy at the time of hospitalization, N (%)	No/unknown	144 (67.0%)	62 (39.5%)	**<0.001** ^**C**^	69 (65.7%)	38 (40.4%)	**<0.001** ^**C**^	61 (69.3%)	19 (38.0%)	**<0.001** ^**C**^	14 (63.6%)	5 (38.5%)	0.179^E^
	Yes	71 (33.0%)	95 (60.5%)		36 (34.3%)	56 (59.6%)		27 (30.7%)	31 (62.0%)		8 (36.4%)	8 (61.5%)	
Presence of ascites at the time of hospitalization, N (%)	No/unknown	55 (25.6%)	12 (7.6%)	**<0.001** ^**C**^	26 (24.8%)	9 (9.6%)	**0.005** ^**C**^	26 (29.5%)	3 (6.0%)	**<0.001** ^**E**^	3 (13.6%)	0 (0.0%)	0.279^E^
	Yes	160 (74.4%)	145 (92.4%)		79 (75.2%)	85 (90.4%)		62 (70.5%)	47 (94.0%)		19 (86.4%)	13 (100.0%)	
Underlying cause of cirrhosis, N (%)	Alcohol-related cirrhosis	147 (68.4%)	83 (52.9%)	**0.002** ^**C**^	65 (61.9%)	43 (45.7%)	**0.022** ^**C**^	66 (75.0%)	28 (56.0%)	**0.021** ^**C**^	16 (72.7%)	12 (92.3%)	0.220^E^
	Hepatitis C	33 (15.3%)	27 (17.2%)	0.632^C^	20 (19.0%)	15 (16.0%)	0.568^C^	10 (11.4%)	10 (20.0%)	0.166^C^	3 (13.6%)	2 (15.4%)	>0.999^E^
	NASH or NAFLD	37 (17.2%)	31 (19.7%)	0.532^C^	20 (19.0%)	21 (22.3%)	0.566^C^	13 (14.8%)	10 (20.0%)	0.428^C^	4 (18.2%)	0 (0.0%)	0.274^E^
	Primary biliary cholangitis	5 (2.3%)	4 (2.5%)	>0.999^E^	5 (4.8%)	2 (2.1%)	0.450^E^	0 (0.0%)	1 (2.0%)	0.362^E^	0 (0.0%)	1 (7.7%)	0.371^E^
	Primary sclerosing cholangitis	1 (0.5%)	2 (1.3%)	0.576^E^	1 (1.0%)	1 (1.1%)	>0.999^E^	0 (0.0%)	1 (2.0%)	0.362^E^	0 (0.0%)	0 (0.0%)	NA
	Other	22 (10.2%)	18 (11.5%)	0.705^C^	15 (14.3%)	11 (11.7%)	0.589^C^	5 (5.7%)	5 (10.0%)	0.496^E^	2 (9.1%)	2 (15.4%)	0.618^E^
Precipitating events, N (%)	Treatment with diuretics	79 (36.7%)	67 (42.7%)	0.247^C^	37 (35.2%)	42 (44.7%)	0.174^C^	33 (37.5%)	20 (40.0%)	0.772^C^	9 (40.9%)	5 (38.5%)	>0.999^E^
	Gastrointestinal bleeding	60 (27.9%)	28 (17.8%)	**0.024** ^**C**^	23 (21.9%)	15 (16.0%)	0.287^C^	27 (30.7%)	10 (20.0%)	0.173^C^	10 (45.5%)	3 (23.1%)	0.282^E^
	Large-volume paracentesis	42 (19.5%)	66 (42.0%)	**<0.001** ^**C**^	17 (16.2%)	39 (41.5%)	**<0.001** ^**C**^	17 (19.3%)	21 (42.0%)	**0.004** ^**C**^	8 (36.4%)	6 (46.2%)	0.568^C^
	Diarrhea	20 (9.3%)	33 (21.0%)	**0.001** ^**C**^	10 (9.5%)	17 (18.1%)	0.078^C^	5 (5.7%)	13 (26.0%)	**0.001** ^**E**^	5 (22.7%)	3 (23.1%)	>0.999^E^
	Other infection/other	29 (13.5%)	16 (10.2%)	0.335^C^	13 (12.4%)	10 (10.6%)	0.701^C^	13 (14.8%)	4 (8.0%)	0.292^E^	3 (13.6%)	2 (15.4%)	>0.999^E^
	None	37 (17.2%)	26 (16.6%)	0.869^C^	18 (17.1%)	19 (20.2%)	0.578^C^	15 (17.0%)	7 (14.0%)	0.639^C^	4 (18.2%)	0 (0.0%)	0.274^E^
Procedures received during first hospitalization, N (%)	TIPS	6 (2.8%)	3 (1.9%)	0.739^E^	0 (0.0%)	1 (1.1%)	0.472^E^	1 (1.1%)	1 (2.0%)	>0.999^E^	5 (22.7%)	1 (7.7%)	0.377^E^
	Renal replacement therapy	23 (10.7%)	44 (28.0%)	**<0.001** ^**C**^	5 (4.8%)	24 (25.5%)	**<0.001** ^**E**^	9 (10.2%)	15 (30.0%)	**0.003** ^**C**^	9 (40.9%)	5 (38.5%)	>0.999^E^
	Liver transplant	0 (0.0%)	19 (12.1%)	**<0.001** ^**E**^	0 (0.0%)	11 (11.7%)	**<0.001** ^**E**^	0 (0.0%)	7 (14.0%)	**<0.001** ^**E**^	0 (0.0%)	1 (7.7%)	0.371^E^
Albumin use, N (%)	No	44 (20.5%)	2 (1.3%)	**<0.001** ^**E**^	22 (21.0%)	1 (1.1%)	**<0.001** ^**E**^	17 (19.3%)	1 (2.0%)	**<0.001** ^**E**^	5 (22.7%)	0 (0.0%)	0.113^E^
	Yes	146 (67.9%)	155 (98.7%)		75 (71.4%)	93 (98.9%)		56 (63.6%)	49 (98.0%)		15 (68.2%)	13 (100.0%)	
	Unknown	25 (11.6%)	0 (0.0%)		8 (7.6%)	0 (0.0%)		15 (17.0%)	0 (0.0%)		2 (9.1%)	0 (0.0%)	
Albumin use during HRS hospitalization (d)	Median (Q1–Q3)	7.0 (4.0–10.0)	7.0 (4.0–12.0)	0.641^W^	7.0 (4.0–11.0)	8.0 (4.0–13.0)	0.571^W^	7.0 (5.0–9.0)	7.0 (4.0–12.0)	0.538^W^	10.0 (4.0–15.0)	4.0 (3.0–11.0)	0.239^W^
Antibiotics, N (%)	No	86 (40.0%)	67 (42.7%)	**<0.001** ^**E**^	43 (41.0%)	42 (44.7%)	**0.003** ^**E**^	37 (42.0%)	19 (38.0%)	0.074^E^	6 (27.3%)	6 (46.2%)	0.554^E^
	Yes	110 (51.2%)	90 (57.3%)		51 (48.6%)	52 (55.3%)		44 (50.0%)	31 (62.0%)		15 (68.2%)	7 (53.8%)	
	Unknown	19 (8.8%)	0 (0.0%)		11 (10.5%)	0 (0.0%)		7 (8.0%)	0 (0.0%)		1 (4.5%)	0 (0.0%)	
Treatment with diuretics on the day before diagnosis, N (%)	No	16 (7.4%)	90 (57.3%)	**<0.001** ^**E**^	15 (14.3%)	52 (55.3%)	**<0.001** ^**E**^	1 (1.1%)	30 (60.0%)	**<0.001** ^**E**^	0 (0.0%)	8 (61.5%)	**<0.001** ^**E**^
	Yes	62 (28.8%)	67 (42.7%)		22 (21.0%)	42 (44.7%)		31 (35.2%)	20 (40.0%)		9 (40.9%)	5 (38.5%)	
	Unknown	137 (63.7%)	0 (0.0%)		68 (64.8%)	0 (0.0%)		56 (63.6%)	0 (0.0%)		13 (59.1%)	0 (0.0%)	

C, Chi-square test; HRS, hepatorenal syndrome; HRS-AKI, HRS-acute kidney injury; E, exact Fisher; NA, not applicable; RRT, renal replacement therapy; TE, equal t-test; TIPS, transjugular intrahepatic portosystemic stent shunt; W, Wilcoxon rank sum.

Bold values indicate statistical significance at *P* < 0.05.

### HRS-AKI treatment

The majority of UK patients (90.2%) received terlipressin, while 89.2% initiated MO in the United States. Only 1.9% patients in the United Kingdom received MO (Table 1). Median daily midodrine dose was 30 mg (range 7.5 mg–90 mg daily). Dose information was not available on octreotide in the US cohort or terlipressin in the UK cohort, respectively. Time to initial vasoconstrictor treatment was longer in the United Kingdom (median 4 vs 2 days, *P* < 0.001). Concomitant albumin was administered in 67.9% of the UK and 98.7% of the US patients. Albumin dosing information was available for the US cohort, with a median total dose of concomitant albumin 180 g (interquartile range 105–303 g) for MO patients. Albumin dosing information was not available for the UK cohort. During the index HRS-AKI hospitalization, 10.7% were treated with RRT in the United Kingdom compared with 28.0% in the United States; none of the UK patients received LT, compared with 12.1% in the United States.

### Treatment outcomes

In unadjusted analysis, patients in the United Kingdom had higher HRS reversal (46.0% vs 14.6%, *P* < 0.001) and on-treatment HRS reversal (47.9% vs 22.3%, *P* < 0.001) as compared with US cohort (Supplemental Table 1, http://links.lww.com/CTG/B420). This trend was preserved when also stratified by SCr severity.

### Renal replacement therapy

Excluding LT recipients, patients who received RRT during their hospitalization in the United Kingdom were more likely to have increased severity and higher SCr at baseline in contrast with the US cohort (Supplemental Table 2, http://links.lww.com/CTG/B420). RRT was more frequently required among patients who did not achieve HRS reversal such that HRS reversal was achieved in only 8.7% vs 6.3% of patients in the UK and US cohorts who received RRT, respectively. In this analysis, RRT occurred after treatment completion and within a 14-day window, representing a distinct episode of AKI after HRS reversal.

### Terlipressin vs midodrine/octreotide: CBPS comparison

**Figure 2.**
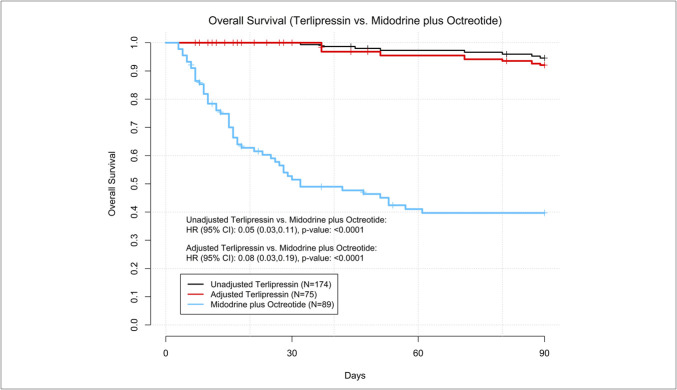
Covariate balancing propensity score-adjusted overall survival for terlipressin vs midodrine plus octreotide.

Baseline differences in the UK and US CBPS cohorts were observed when comparing those who received terlipressin (N = 174) vs MO (N = 89) (Table 2). After adjustment, the baseline characteristics were well balanced between 2 groups (Table 2), with an effective weighted sample size of N = 75 for the matched terlipressin group. In adjusted analysis, real-world use of terlipressin resulted in higher HRS reversal than patients receiving MO (53.2% vs 16.9% [mean difference of 36.3%, *P* < 0.0001]) (Table 3). Secondary endpoints were also more favorable for patients on terlipressin, including adjusted on-treatment HRS reversal (54.2% vs 23.6%, [mean difference of 30.6%, *P* < 0.0001]). Adjusted SCr reduction from baseline to last day of treatment was −1.00 mg/dL for terlipressin vs no change (0.08 mg/dL) for MO (*P* < 0.0001) (Table 3). Adjusted OS was more favorable among patients treated with terlipressin vs MO (HR 0.08; 95% CI 0.03, 0.11; *P* < 0.0001; Figure 2). A sensitivity analysis not adjusting for ascites nor encephalopathy had little impact on study results for weighted HRS reversal and on-treatment HRS reversal.

**Table 2.** Covariate balancing propensity score cohort baseline characteristics: terlipressin (the United Kingdom) vs midodrine plus octreotide (the United States)

Variable description	Statistic or category	CBPS adjusted			Unadjusted		
		Terlipressin (the United Kingdom) (N = 75)	Midodrine and octreotide (the United States) (N = 89)	*P* value	Terlipressin (the United Kingdom) (N = 174)	Midodrine and octreotide (the United States) (N = 89)	*P* value
Duration of initial therapy	Median (Q1–Q3)	5.27 (3.91–7.62)	5.90 (2.71–9.69)	0.109	5.91 (4.06–8.65)	5.90 (2.71–9.69)	0.563
Time to initial treatment (d)	Median (Q1–Q3)	3.65 (2.18–7.75)	1.83 (1.00–4.25)	**0.001**	3.50 (1.97–7.72)	1.83 (1.00–4.25)	**<0.001**
Age^a^	Mean (SD)	57.12 (16.73)	57.12 (12.60)	>0.999	53.21 (11.51)	57.12 (12.60)	**0.015**
	Median (Q1–Q3)	57.68 (48.81–63.31)	60.75 (49.04–66.11)		52.40 (45.21–61.50)	60.75 (49.04–66.11)	
	Range	29.00 to 77.00	28.00 to 79.00		29.00 to 77.00	28.00 to 79.00	
Sex, N (%)^a^	Male	55.06%	55.06%	>0.999	115 (66.09%)	49 (55.06%)	0.081
	Female	44.94%	44.94%		59 (33.91%)	40 (44.94%)	
SCr level in mg/dL at baseline	Mean (SD)	2.97 (1.69)	2.85 (1.02)	0.488	3.22 (1.54)	2.85 (1.02)	**0.023**
	Median (Q1–Q3)	2.57 (2.09–3.50)	2.54 (2.06–3.32)		2.82 (2.12–3.82)	2.54 (2.06–3.32)	
	Range	1.51 to 10.62	1.57 to 6.51		1.51 to 10.62	1.57 to 6.51	
SCr severity, N (%)^a^	Mild (≤3)	64.04%	64.04%	>0.999	92 (52.87%)	57 (64.04%)	0.187
	Moderate (>3 & <5)	31.46%	31.46%		67 (38.51%)	28 (31.46%)	
	Severe (≥5)	4.49%	4.49%		15 (8.62%)	4 (4.49%)	
Presence of encephalopathy at the time of hospitalization, N (%)^a^	No/unknown	44.94%	44.94%	>0.999	122 (70.11%)	40 (44.94%)	**<0.001**
	Yes	55.06%	55.06%		52 (29.89%)	49 (55.06%)	
Presence of ascites at the time of hospitalization, N (%)^a^	No/unknown	6.74%	6.74%	>0.999	41 (23.56%)	6 (6.74%)	**<0.001**
	Yes	93.26%	93.26%		133 (76.44%)	83 (93.26%)	
Albumin use, N (%)^a^	No	0.00%	0 (0.00%)	>0.999	33 (18.97%)	0 (0.00%)	**<0.001**
	Unknown	0.00%	0 (0.00%)		18 (10.34%)	0 (0.00%)	
	Yes	100.00%	100.00%		123 (70.69%)	89 (100.00%)	
Albumin use during HRS hospitalization (d)^a^	Mean (SD)	7.29 (4.85)	7.29 (5.55)	>0.999	8.47 (6.47)	7.29 (5.55)	0.156
	Median (Q1–Q3)	5.70 (3.83–7.95)	4.94 (2.59–9.88)		6.38 (4.06–9.36)	4.94 (2.59–9.88)	

CBPS, covariate balancing propensity score; HRS, hepatorenal syndrome; SCr, serum creatinine.

Bold values indicate statistical significance at *P* < 0.05.

a These patient characteristics were adjusted for using CBPS.

**Table 3.** Covariate balancing propensity score cohort treatment response: terlipressin vs midodrine plus octreotide

Variable description	CBPS adjusted				Unadjusted			
	Terlipressin (ESS = 75)^a^	Midodrine & octreotide (N = 89)	Mean difference (95% CI)	*P* value	Terlipressin (N = 174)	Midodrine & octreotide (N = 89)	Mean difference (95% CI)	*P* value
HRS reversal, %	53.17%	16.85%	36.31 (22.44, 50.18)	**<0.0001**	52.30%	16.85%	35.44 (24.60, 46.29)	**<0.0001**
HRS reversal or partial response, %	74.10%	24.72%	49.38 (35.09, 63.67)	**<0.0001**	72.41%	24.72%	47.69 (36.45, 58.94)	**<0.0001**
On-treatment HRS reversal, %	54.18%	23.60%	30.59 (16.08, 45.10)	**<0.0001**	54.60%	23.60%	31.00 (19.39, 42.61)	**<0.0001**
Change in kidney function (SCr) from baseline (mg/dL), mean (SD)	−1.00 (1.79)	0.08 (1.35)	−1.08 (0.69,1.46)	**<0.0001**	−1.00 (1.71)	0.08 (1.35)	−1.08 (0.70, 1.46)	**<0.0001**
Percent improvement in kidney function (SCr) from baseline, mean (SD)	34.12 (55.60)	−7.85 (54.95)	41.96 (27.82, 56.12)	**<0.0001**	31.43 (43.98)	−7.85 (54.95)	39.28 (26.07, 52.48)	**<0.0001**

CBPS, covariate balancing propensity score; EES, effective sample size; HRS, hepatorenal syndrome; SCr, serum creatinine.

a ESS = 75 is the ESS for the terlipressin group after CBPS reweighting.

Bold values indicate statistical significance at *P* < 0.05.

## DISCUSSION

The comparative analysis of HRS-AKI management in the United Kingdom and the United States using patient-level data confirms that terlipressin was a more effective treatment option in achieving HRS-AKI reversal in the real world. After adjustment for observable baseline characteristics, patients who were treated with terlipressin in the United Kingdom were more likely to exhibit improved kidney function than those receiving MO in the United States.

Our study findings confirm previous reports that showed MO treatment to be ineffective at achieving HRS reversal, including a prospective randomized clinical trial by Cavallin et al, (18) which was prematurely terminated because of the risk of nonresponse among patients receiving MO compared with terlipressin. The effect sizes for treatment response associated with terlipressin vs MO identified in this study, including reduction in SCr and achievement of HRS reversal, are consistent with previous reports (12,18). The adjusted HRS reversal with terlipressin in our real-world UK cohort, defined by SCr <1.5 mg/dL at end of therapy, was 53.2%, similar to the 48.2% observed response in the study of Gonzalez et al (adjusted on-treatment HRS reversal comparison of 54.2% vs 52.4% between studies) which used terlipressin data from the CONFIRM/REVERSE trials, and where baseline differences including MELD score, ACLF grade, and bilirubin could be adjusted for (12). Moreover, the adjusted difference in HRS reversal between the real-world use of terlipressin vs real-world use of MO in this study was similar (36.3% vs 33.7%) to the study of Gonzalez et al (12) as were absolute change and percent improvement in kidney function. These data add to an increasing body of evidence demonstrating poor efficacy of MO in the treatment of HRS-AKI and unequivocally support the use of terlipressin as first-line therapy in the United States based on real-world evidence.

The contrast between patients in the UK and US cohorts is clear. Although patients with HRS-AKI from the United Kingdom may be expected to have a better prognosis than those in the United States given their younger age and a lower prevalence of encephalopathy/ascites, they also started from a higher baseline SCr. Additional differences were observed, including a longer time to initial treatment in the UK cohort; however, this may reflect the confidence of UK physicians to be able to reverse HRS-AKI with terlipressin if other treatments fail rather than delayed HRS-AKI diagnosis. Consequently, this characteristic was not included for adjustment nor was HRS-AKI hospitalization setting because the definition of intensive vs nonintensive care is different between the 2 countries. Instead, adjusting for baseline clinical characteristics including SCr was considered sufficient, given the established impact of early diagnosis and baseline SCr level on response (13,19,20).

Guidance on the use of albumin in the setting of AKI and during the course of vasoconstrictor therapy for HRS-AKI has evolved, highlighting the importance of evaluating an individual patient's intravascular volume status. Existing guidance has recommended the administration of albumin to achieve a diagnosis of HRS-AKI and to enhance treatment response when used concurrently with terlipressin (2,21,22). However, the benefit of albumin may be greatest among individuals with evidence of intravascular volume depletion, while caution should be exercised to avoid complications such as pulmonary edema among those with risk of volume overload (11,23,24). In our study, the use of albumin was notably lower in the UK cohort. This observation may reflect a greater experience with using terlipressin and albumin among UK physicians, and confidence of expected efficacy. Thus, US physicians may continue giving more albumin simply because MO is not effective. Of note, the UK study also reported a significant number of respiratory complications, calling attention to the potential risk of pulmonary edema in the setting of intravascular volume overload and terlipressin therapy. Furthermore, some hospitals have established restrictions on albumin use, encouraging a conservative approach (25,26).

Although strengths of our study include the use of patient-level data and adjustment methods, systemic differences between the UK and US cohorts may exist despite similar medical education among UK and US physicians. For example, the UK healthcare system is funded by the state, and the only pressure to discharge early is based on bed availability rather than cost. This is reflected in a longer length of stay during the HRS-AKI hospitalization of 27 days for UK patients vs 11 days for the US cohort, respectively.

The application of differential exclusion criteria during patient selection could affect survival comparisons despite the use of similar study protocols across the 2 chart reviews. Specifically, patients with prior dialysis or TIPS within 1 month of HRS-AKI hospitalization, had prior liver transplantation, required hospitalization for HRS-AKI during the previous 6 months, and those who died within 24 hours of vasopressor initiation were excluded from the UK chart review. Moore et al (13) previously reported on the possibility of survival bias in the UK data in which more severe cases or cases with incomplete data were excluded. Any of these factors could contribute to a higher adjusted OS observed in the terlipressin group (Figure 2). Therefore, survival outcomes in this study should be interpreted with caution. In addition, the time frames when data were collected for the 2 chart reviews overlapped with the updated definition of HRS-AKI by the International Club of Ascites in 2015 (27), in which the UK cohort could have mixed HRS-AKI cases (hospitalized between 2013 and 2017), whereas the US cohort (hospitalized between 2016 and 2019) included cases uniformly meeting the updated HRS-AKI definition. As changes in SCr define AKI stage and HRS-AKI, limitations also exist in the precise determination of kidney function in the setting of cirrhosis and portal hypertension, especially among those with recurrent ascites and sarcopenia in whom SCr may overestimate kidney function (28). Efforts to identify more accurate markers of kidney function in this high-risk population will be essential in early recognition of HRS-AKI and optimization of treatment outcomes with terlipressin.

A challenge encountered in this study was the missing date of initiation of RRT among patients in the UK cohort. RRT is usually initiated after vasoconstrictor treatment failure is established. To understand the potential effect of timing of RRT on SCr values, treatment response was investigated separately in the UK and US data sets based on whether RRT was initiated during the HRS-AKI hospitalization. This analysis found that treatment response was similarly low in the United Kingdom and the United States among patients who received RRT (8.7% vs 6.3%, respectively) (Supplemental Table 2, http://links.lww.com/CTG/B420). Given the equally low treatment response among patients who initiated RRT between the 2 cohorts, as well as a further inquiry confirming that RRT use in the United States occurred after the completion of vasoconstrictor treatment, we concluded that the lack of date for RRT administration among UK patients would not materially bias the analysis. As no patients in the UK cohort underwent LT, similarly investigating the role of LT among patients in the UK vs US cohorts was not necessary. In addition, specific dosing information was inconsistent between the US and UK cohorts, including the unavailability of octreotide dosing in the US cohort and both terlipressin and albumin dosing in the UK cohort.

Available data regarding terlipressin uptake and utilization among US hospitals after US Food and Drug Administration approval in 2022 reveal that the majority of individuals who receive terlipressin in the United States are hospitalized at large academic medical centers (16). Several factors may contribute to this observation including terlipressin availability, early safety concerns, and the complexity of the patient population requiring on-treatment monitoring. Consequently, most patients diagnosed with HRS-AKI in the United States continue to receive MO despite its low efficacy (18). In addition, unresolved questions remain, including HRS-AKI treatment strategies among US LT candidates (29). As clinician experience with patient selection, management of HRS-AKI, and terlipressin availability expand in the United States, increased utilization of terlipressin as the standard of care for HRS-AKI is anticipated.

This post hoc indirect comparison of real-world data from 2 medical chart reviews demonstrated that terlipressin, the first-line treatment for HRS-AKI in the United Kingdom, improved kidney function more effectively than MO, the most commonly used off-label treatment in the United States. These findings are consistent with other studies and our previous report comparing outcomes in a US population including patients enrolled in the CONFIRM/REVERSE trials who received terlipressin vs patients treated with MO based on chart review (12). The addition of real-world evidence to published clinical trials provides valuable perspective to further support proposed guidelines indicating terlipressin as the first-line treatment of HRS-AKI in the United States.

## CONFLICTS OF INTEREST

**Guarantor of the article:** Stevan A. Gonzalez, MD, MS.

**Specific author contributions:** A.S.A., K.M., S.A.G., D.A.S., V.C., X.H.: study concept and design; V.C., X.H., K.M.: acquisition of data; A.S.A., K.M., S.A.G., D.A.S., V.C., W.J.W., X.H.: analysis and interpretation of data; V.C., W.J.W.: drafting of the manuscript; A.S.A., K.M., S.A.G., D.A.S., V.C., W.J.W., X.H.: critical revision of the manuscript for important intellectual content; V.C., W.J.W.: statistical analysis; V.C., X.H.: obtained funding; V.C., X.H.: administrative, technical, or material support; V.C., X.H.: study supervision. All authors approved the final manuscript version submitted.

**Financial support:** This study was funded by Mallinckrodt Pharmaceuticals.

**Potential competing interests:** A.S.A. has consulted for Mallinckrodt Pharmaceuticals, Ocelot Bio, Motric Bio, Sequana Medical, and Bioporto. A.S.A. was funded by NIH award K23 DK128567. K.M. has consulted for Mallinckrodt Pharmaceuticals. S.A.G. is a speakers' bureau member and has consulted for Mallinckrodt Pharmaceuticals. D.A.S. has consulted for BioVie, Evive, and Mallinckrodt. V.C. is an employee of OPEN Health, which received funding to assist in the conduct of this study. W.J.W. was an employee of Open Health at the time of the study. X.H. is an employee of Mallinckrodt Pharmaceuticals and may own stock options. K.M. has been paid as a consultant to Mallinckrodt Pharmaceuticals in the last 5 years.

**Data sharing statement:** Additional information and materials on the study may be provided upon reasonable request to authors.

**Study Highlights**

**WHAT IS KNOWN**

✓ Hepatorenal syndrome-acute kidney injury (HRS-AKI) is a severe form of AKI that develops in patients with advanced cirrhosis.
✓ Untreated HRS-AKI can result in irreversible kidney failure, with >80% mortality at 3 months.
✓ For first-line treatment, guidelines recommend terlipressin, a synthetic vasopressin receptor agonist, along with albumin.
✓ Although approved in Europe over 2 decades ago, terlipressin was only recently approved in the United States.
✓ US historic standard of care is midodrine and octreotide (MO).

**WHAT IS NEW HERE**

✓ Terlipressin improved kidney function more effectively than MO in a real-world effectiveness comparison.
✓ Our study confirms previous findings showing MO treatment to be ineffective at achieving HRS reversal.
✓ Real-world evidence suggests that terlipressin could benefit many patients diagnosed with HRS-AKI.

## Supplementary Material

## ABBREVIATIONS:

ACLF: acute-on-chronic liver failure
CBPS: covariate balancing propensity score
HRS-AKI: hepatorenal syndrome-acute kidney injury
ICD: International Classification of Diseases
IRB: Institutional Review Board
LT: liver transplant
MELD: Model for End-Stage Liver Disease
MO: midodrine plus octreotide
OS: overall survival
RRT: renal replacement therapy
SCr: serum creatinine
