# Supplementary material for: Real-World Indirect Treatment Comparison of Terlipressin vs Midodrine Plus Octreotide in Hepatorenal Syndrome-Acute Kidney Injury
Source: Clin Transl Gastroenterol. 2025 Nov 20;17(2):e00951. doi: 10.14309/ctg.0000000000000951 (PMC12922934; doi:10.14309/ctg.0000000000000951)
Supplement: Supplementary file 1 [file ct9-17-e00951-s001.docx]

# **Supplemental Materials**

**Real-World Indirect Treatment Comparison of Terlipressin Versus Midodrine Plus Octreotide in Hepatorenal Syndrome-Acute Kidney Injury**

**Supplemental Table 1. Overall treatment response by level of SCr among HRS patients in the UK and US**

|  |  | **All patients** | | | **Mild (≤3mg/dL)** | | | **Moderate (>3 & <5mg/dL)** | | | **Severe (≥5 mg/dL)** | | |
| --- | --- | --- | --- | --- | --- | --- | --- | --- | --- | --- | --- | --- | --- |
| **Variable** | **Statistic or category** | **UK**  **(N = 215** | **US**  **(N = 157)** | ***P* value** | **UK**  **(N = 105)** | **US**  **(N = 94)** | ***P* value** | **UK**  **(N = 88)** | **US**  **(N = 50)** | ***P* value** | **UK**  **(N = 22)** | **US**  **(N = 13)** | ***P* value** |
| **HRS response on the last day of treatment, N (%)** | HRS reversal | 99 (46.0%) | 23 (14.6%) | **<0.001** | 76 (72.4%) | 17 (18.1%) | <0.001 | 20 (22.7%) | 6 (12.0%) | **<0.001** | 3 (13.6%) | 0  (0.0%) | **0.004** |
|  | Partial Response | 45 (20.9%) | 9 (5.7%) |  | 2 (1.9%) | 3 (3.2%) |  | 32 (36.4%) | 5 (10.0%) |  | 11 (50.0%) | 1 (7.7%) |  |
|  | No Response | 71 (33.0%) | 125 (79.6%) |  | 27 (25.7%) | 74 (78.7%) |  | 36 (40.9%) | 39 (78.0%) |  | 8 (36.4%) | 12 (92.3%) |  |
| **On-treatment HRS reversal, N (%)** | Yes | 103 (47.9%) | 35 (22.3%) | **<0.001** | 77 (73.3%) | 28 (29.8%) | **<0.001** | 22 (25.0%) | 7 (14.0%) | 0.127 | 4 (18.2%) | 0  (0.0%) | 0.274 |
| **Change in SCr from baseline to day 14 or to the day of treatment discontinuation, whichever occurred first** | Mean (SD) | -0.99 (1.72) | 0.19 (1.38) | **<0.001** | -0.62 (1.16) | 0.35 (1.26) | <0.001 | -1.02 (1.52) | -0.04 (1.55) | **<0.001** | -2.63 (3.21) | -0.10 (1.48) | **0.003** |
| **Relative change (%) in SCr from baseline to day 14 or to the day of treatment discontinuation, whichever occurred first** | Mean (SD) | 28.29 (44.93) | -11.48 (55.96) | **<0.001** | 27.99 (50.05) | -19.76 (63.79) | <0.001 | 27.17 (38.45) | 0.51 (41.79) | **<0.001** | 34.23 (44.73) | 2.23 (26.59) | **0.026** |

HRS, hepatorenal syndrome; SCr, serum creatinine.

Bold values indicate statistical significance at *P* < 0.05.

**Supplemental Table 2. Overall comparison of treatment response achieved by RRT vs non-RRT patients during HRS-AKI hospitalization**

|  | | **UK (excluding liver transplant recipients)** | | | **US (excluding liver transplant recipients)** | | |
| --- | --- | --- | --- | --- | --- | --- | --- |
| **Variable** | **Statistic or category** | **Non-RRT recipients, during hospitalization**  **(N = 192)** | **RRT recipients, during hospitalization**  **(N = 23)** | ***P*value** | **Non-RRT recipients, during hospitalization**  **(N = 106)** | **RRT recipients, during hospitalization**  **(N = 32)** | ***P*value** |
| **Initiation treatment,**  **N (%)** | Midodrine/Octreotide | 4 (2.1%) | 0 (0.0%) | 0.749 | 94 (88.7%) | 27 (84.4%) | 0.715 |
|  | Norepinephrine/Noradrenaline | 4 (2.1%) | 0 (0.0%) |  | 4 (3.8%) | 2 (6.3%) |  |
|  | Terlipressin | 171 (89.1%) | 23 (100.0%) |  | 0 (0.0%) | 0 (0.0%) |  |
|  | Vasopressin | 13 (6.8%) | 0 (0.0%) |  | 0 (0.0%) | 0 (0.0%) |  |
| **SCr level in mg/dL at baseline** | N | 192 | 23 | **<0.001** | 106 | 32 | 0.908 |
|  | Mean (SD) | 3.13 (1.32) | 5.08 (2.62) |  | 2.95 (1.14) | 3.13 (1.48) |  |
|  | Median (Q1 to Q3) | 2.8 (2.1 to 3.7) | 4.3 (3.1 to 7.6) |  | 2.6 (2.1 to 3.5) | 2.8 (2.0 to 3.7) |  |
|  | Range | 1.5 to 8.9 | 1.6 to 10.6 |  | 1.5 to 6.3 | 1.6 to 7.3 |  |
| **SCr severity,**  **N (%)** | Mild (≤3 mg/dL ) | 100 (52.1%) | 5 (21.7%) | **<0.001** | 65 (61.3%) | 18 (56.3%) | 0.642 |
|  | Moderate (>3 & <5 mg/dL) | 79 (41.1%) | 9 (39.1%) |  | 33 (31.1%) | 10 (31.3%) |  |
|  | Severe (≥5 mg/dL) | 13 (6.8%) | 9 (39.1%) |  | 8 (7.5%) | 4 (12.5%) |  |
| **Response on the last day of treatment*, N (%)** | a. HRS reversal | 97 (50.5%) | 2 (8.7%) | **<0.001** | 20 (18.9%) | 2 (6.3%) | 0.130 |
|  | b. Partial response | 40 (20.8%) | 5 (21.7%) |  | 8 (7.5%) | 1 (3.1%) |  |
|  | c. No response | 55 (28.6%) | 16 (69.6%) |  | 78 (73.6%) | 29 (90.6%) |  |
| **On-treatment HRS reversal,**  **N (%)** | Yes | 100 (52.1%) | 3 (13.0%) | **<0.001** | 30 (28.3%) | 3 (9.4%) | **0.033** |

HRS, hepatorenal syndrome; RRT, renal replacement therapy; SCr, serum creatinine.

Bold values indicate statistical significance at *P* < 0.05.
